# Effect of addition of dried potato pulp on selected quality characteristics of shortcrust pastry cookies

**DOI:** 10.1515/biol-2020-0093

**Published:** 2020-10-07

**Authors:** Hanna Boruczkowska, Tomasz Boruczkowski, Danuta Figurska-Ciura, Wioletta Drożdż

**Affiliations:** Department of Food Storage and Technology, Wrocław University of Environmental and Life Sciences, Wrocław, Poland; Department of Human Nutrition, Wrocław University of Environmental and Life Sciences, Wrocław, Poland

**Keywords:** dietary fiber, potato pulp, color, consistency

## Abstract

Potato pulp is a nuisance waste from the production of potato starch, containing large amounts of dietary fiber; therefore, addition of the pulp to food may have a positive effect on the nutritional value of food products. To increase the amount of dietary fiber, shortcrust pastry cookies were baked by replacing some of the flour (20–100%) with an equivalent amount of dried potato pulp. In all the tested variants, correct confectionery products were obtained. The color of finished product (measured using Konica-Minolta CM-5 spectrophotometer) and mechanical properties of raw dough and baked cookies (subjected to a tensile test, using INSTRON 5544 Tensile Tester) were determined. Furthermore, the samples were subjected to sensory consumer assessment (evaluated on a 7-point hedonic scale). Colorimetric tests of the cookies showed a slight shift in their color from yellow toward green but at the same time lowering its saturation. Strength tests show that only the replacement of more than 40% of flour by the dried pulp resulted in a significant increase in the hardness of baked products. Regarding the tests with consumers, cookies containing up to 40% potato pulp were acceptable. Such a large addition of potato pulp to shortcrust pastry cookies creates new opportunities for this waste management.

## Graphical abstract

**Figure:**
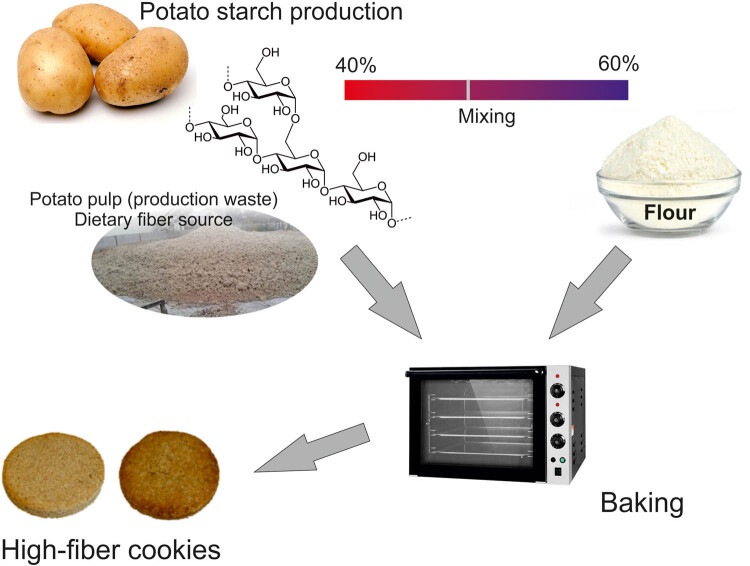

## 1 Introduction

Shortcrust pastry products are tasty, and hence, their consumption is high. The usual proportions of the major ingredients are: three parts of flour, two parts of fat, and one part of sugar, but they can deviate slightly to achieve the preferred final effect. For the production of shortcrust pastry, usually refined flour is used, although it is devoid of many important nutrients [1]. Thanks to the increasing consumer awareness, most of the staple foods are enriched with bioactive compounds, which stimulate human metabolism [2,3]. The health-promoting compounds include oligosaccharides, fatty polyols, amino acids, peptides, proteins, polyunsaturated fatty acids, vitamins, and dietary fiber [4].

According to the definition proposed by the European Food Safety Authority, dietary fiber consists of carbohydrate polymers with three or more monomeric units, which are neither digested nor absorbed in the human small intestine [5]. In many staple foods, its content is low, so the diet must be supplemented with dietary fiber. Its main sources are cereal products and vegetables [6]. High dietary fiber content is the characteristic of some food industry by-products, such as pressed sugar beet pulp (from sugar production) and dewatered potato pulp (from potato starch production). Potato pulp and potato juice are the by-products of the starch industry. Potato juice constitutes the source of proteins and other bioactive components that have antioxidant properties. Nowadays, successful attempts are engaged to apply processed potato juice into functional food, which is helpful in gastric ailment [7,8,9]. Due to the presence of dietary fiber in potato pulp, the by-product is used so far as fertilizer or animal feed, and this increased significantly the research activity of several authors to find the benefit for the environment [10]. Dietary fiber produced from the dried potato pulp is used primarily in the meat industry [11,12,13,14] and also in bakeries [15]. In the starch industry, by-products constitute about 80% of the mass of raw material, which places it in the third place in Poland among the food industries [16]. For this reason, each subsequent method of using starch waste products gives a chance for their effective management, while reducing the burden of them on the environment [10,17].

This study aimed to assess the quality of shortcrust pastry cookies prepared using a basic recipe in which several percentages of wheat flour was replaced with dried potato pulp [i.e., 20%, 40%, 50%, 60%, and 100% (Table 1)]. The cookies were subjected to physical and organoleptic tests and compared with the control product prepared without the dried potato pulp.

**Table 1:** Tested recipes of shortcrust pastry cookies

Ingredient	Proportion of dried potato pulp in the flour used (%)					
	0	20	40	50	60	100
Wheat flour type 500 (g)	250	200	150	100	50	0
Dried potato pulp (g)	0	50	100	150	200	250
Margarine (g)	125	125	125	125	125	125
Granulated white sugar (g)	50	50	50	50	50	50
Egg yolks (number)	2	2	2	2	2	2
Cream, 18% fat (g)	50	50	50	50	50	50
Baking powder (g)	5	5	5	5	5	5
Vanillin sugar (g)	5	5	5	5	5	5
Water (mL)	0	40	60	80	100	120

## 2 Materials and methods

### 2.1 Sample preparation

Shortcrust pastry cookies were prepared according to a basic recipe (without the addition of dried potato pulp), and next, the recipe was modified so that a part or all of the wheat flour was replaced by an equivalent amount of dried potato pulp (Table 1).

In the experiment, commercially available products were used: refined coarse-ground wheat flour type 500 (in Polish “Krupczatka”, manufactured by LUBELLA Ltd., Lublin), margarine Kasia (Unilever de Argentina S.A.), granulated white sugar (Krajowa Spółka Cukrowa S.A.), fresh eggs (size M, Jajolux Ltd., Sokołowice), 18% cream (Regional Dairy Cooperative, Piątnica), as well as baking powder and vanillin sugar (Winiary S.A.). Dewatered potato pulp, obtained from Przedsiębiorstwo Przemysłu Ziemniaczanego S.A. in Niechlów, was dried to 91.03% dry matter content. The dried pulp had a starch content of 34.2% (measured with the polarimetric method using calcium chloride) and total dietary fiber content of 52.7%, soluble dietary fiber 11.3% and insoluble dietary fiber 41.4% (measured with Total dietary fibre Kit, Merck).

The cookies were baked in three replicates in the laboratory in a combi steamer (Self Cooking Center, Rational, Germany) under controlled conditions: at 210°C in dry air (relative humidity set to 0%). The cookies were gradually cooled down to room temperature, and then, they were placed in plastic bags to prevent drying.

### 2.2 Color measurement

Color was measured using Konica-Minolta CM-5 spectrophotometer. Results were expressed in the CIE L*a*b* color space defined by the International Commission on Illumination in 1976. It expresses color as three values: L* for the lightness from black to white, a* from green to red, and b* from blue to yellow. Furthermore, the hue angle (*H*) and chroma (*C*) of samples, which reflect color perception by the human eye, were calculated. The calculations were made using the formulas given in Konica-Minolta’s user manual [18]. The study was conducted in five replications.

### 2.3 Mechanical properties

The raw dough cut into cookies (circles 50 mm in diameter and 5 mm thick) as well as the baked cookies after cooling down were subjected to the tensile test using INSTRON 5544 Tensile Tester with software version 5.11. The unbaked and baked cookies were cut with a single shear blade (head movement rate 100 mm/min). A head with a measurement range of up to 100 N was used for the measurements. Based on the measurements, the maximum force *F*_max_ (N) and energy *E* (mJ) needed to cut the cookie [19,20] were calculated. The final result of tensile tests is a mean of five replications.

### 2.4 Organoleptic assessment

The baked cookies were also subjected to organoleptic consumer assessment, taking into account their shape, cohesion, and structure, as well as taste and smell of the cookies. All the attributes were evaluated on a 7-point hedonic scale, from 1 (“definitely dislike”) to 7 (“definitely like”), by a group of 65 consumers (55 females and 10 males, aged 22–65 years, with secondary or tertiary education). The evaluations were conducted in a sensory laboratory.

### 2.5 Statistical analysis

The results of instrumental tests were subjected to the statistical analysis using STATISTICA 13 software: one-way analysis of variance and distinguishing of homogeneous groups by the Duncan test at the significance level of *α* = 0.05.

## 3 Results and discussion

### 3.1 Results of color measurements

Consumers are used to the typical appearance of shortbread. Their enrichment with dietary fiber by adding potato pulp to the recipe has a significant impact on their appearance and tenderness. In the colorimetric analysis, the hue angle (*H*) ranged from about 73° in the control cookies to about 77° in the cookies with the maximum amount of dried potato pulp (Table 2). The measured values denote small changes in color: from yellow toward green. It is noteworthy that already the 20% addition of dried potato pulp caused a significant change in cookie hue, but replacement of more than 40% of flour with dried potato pulp did not cause any further change in this parameter.

**Table 2:** Dependence of cookie hue and color saturation value on potato pulp content (% of flour replaced by dried pulp). Homogeneous groups were determined by the Duncan test and marked by small letters, LSD = 0.97

Potato pulp (%)	0%	20%	40%	50%	60%	100%	
Hue (°)	73.18^c^	75.27^b^	76.1^ab^	74.0^c^	76.53^a^	76.71^a^	LSD = 0.97
Color saturation	35.96^a^	31.57^b^	27.26^c^	28.81^d^	25.29^e^	22.92^f^	LSD = 0.97

Much greater changes were observed in values of color saturation (*C*) of the cookies depending on the content of potato pulp. Increasing the content of dried potato pulp caused a significant decrease in color saturation, from 36 for control cookies to 23 in those where flour was completely replaced by the dried potato pulp. Gómez et al. [21] also reported that cookies have lower value of color saturation with the increasing level of chickpea flour. However, the opposite relationship was found by Singh et al. [22], and there was an increase in *C* and a decrease in hue angle in cookies with increasing amounts of sweet potato flour.

**Figure 1:**
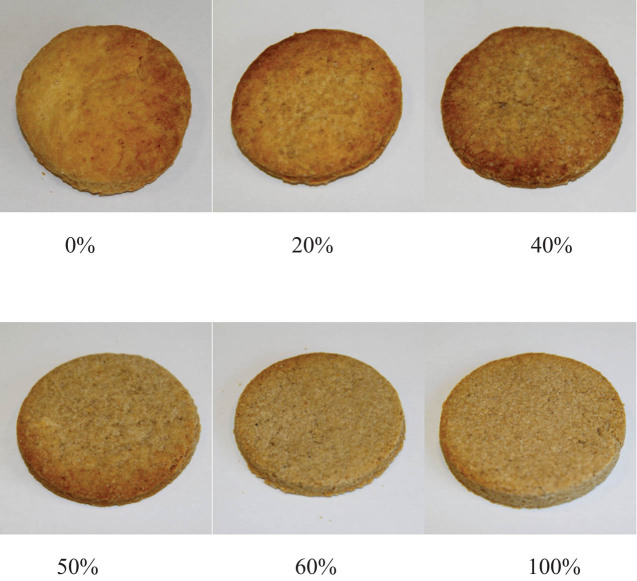
Photographs of baked cookies with 0% to 100% of flour replaced by dried potato pulp.

Changes in cookie color can influence the behavior of consumers. Although the shift of color towards green was not conspicuous, the lower color saturation of cookies with potato pulp may discourage consumers from buying them (Figure 1).

### 3.2 Results of mechanical properties tests

The maximum force *F*_max_ and energy *E* (Table 3) needed to cut the raw dough or the baked cookie also depended on the content of the dried potato pulp. The increasing content of the dried potato pulp was linked with a statistically significant rise in maximum force needed to cut the cookies (from about 4 N for the control to about 16 N for 60% and 100% of flour replaced by potato pulp). The changes in energy needed to cut dough were somewhat different. In dough with potato pulp replacing 40% and 100% of flour, *E* values were only slightly higher than the control value. In contrast, cutting the dough with 60% of potato pulp required the greatest energy (about 16 mJ). The presented results indicate that the increased proportion of potato pulp caused the formation of a harder surface layer on the dough, and the cohesion of the inner part of the dough was most similar to the control in the case of dough with 40% and 100% of flour replaced by the dried potato pulp.

**Table 3:** Dependence of the maximum force needed to cut the raw dough and the cookies (*F*_max_) and the energy needed to cut the raw dough and the cookies (*E*) on potato pulp content (% of flour replaced by dried pulp). Homogeneous groups were determined by the Duncan test and marked by small letters

Potato pulp (%)	0%	20%	40%	50%	60%	100%	
*F*_max_ (N) of raw dough	3.44^a^	7.05^b^	8.96^bc^	10.73^c^	15.4^d^	14.12^d^	LSD = 1.92
*E* (mJ) of raw dough	9.1^a^	16.56^b^	13.24^c^	19.79^d^	23.34^e^	12.02^c^	LSD = 2.46
*F*_max_ (N) of cookies	25.26^a^	31.77^ab^	28.78^ab^	53.83^abc^	68.18^bc^	90.31^c^	LSD = 38.6
*E* (mJ) of cookies	11.81^a^	27.67^abc^	23.84^ab^	42.6^bc^	52.0^c^	125.12^d^	LSD = 26.5

Results of strength tests of baked cookies were slightly different (Table 3). Maximum forces needed to cut the cookies with 20–50% of flour replaced by potato pulp were similar and did not differ significantly from the control value. This means that consumer assessment of cookie brittleness after addition of up to 50% of dried potato pulp should be similar.

A similar relationship was observed for measurements of the energy needed to cut the baked cookies: it was the lowest for control cookies (about 12 mJ), but cookies with 20–50% of flour replaced by potato pulp formed a statistically homogeneous group, with energy values varying from 24 to 41 mJ. However, complete replacement of flour with the pulp resulted in an 8-fold increase in the energy needed to cut the cookie. The strength and the energy needed to break or bite cookies are some of the major parameters affecting the acceptance of a food product by consumers [23].

### 3.3 Results of organoleptic assessment

**Figure 2:**
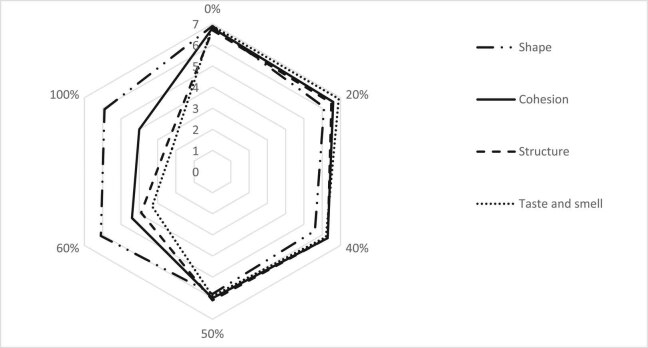
Results of consumer assessment of cookies with 0–100% of flour replaced by dried potato pulp.

To assess how the consumers perceive the texture of the tested cookies, the products were subjected to sensory consumer assessment on a 7-point hedonic scale (Figure 2).

In the sensory tests, control cookies were rated the highest. Increasing proportions of potato pulp resulted in lower scores for cookie structure, taste and smell, and cohesion (consistency). The structure of cookies with potato pulp was rated lower because it was more compact and not typical of shortcrust pastry according to consumers. This problem might be partly solved by longer baking of the cookies with higher potato pulp content. The sensory properties can be affected by the addition of dietary fiber that is present in potato pulp. Many authors have noticed that cookies with a high fiber content are not well accepted [24,25,26,27]. However, there are also studies in which it was found that substituting wheat flour with fiber at levels of up to 40% does not affect consumer acceptability [28]. The study by Jeddou et al. [29] showed that substituting wheat flour in the cake formulation with potato peel powder (up to 10%) maintained the sensory characteristics of the product. Singh et al. [22] observed that incorporation of 40% sweet potato flour yielded approximately similar results compared with wheat flour cookies. Similarly, in our study, the results of consumer assessment as well as measurements of mechanical parameters of the cookies indicate that the replacement of up to 40% of flour by the pulp enables manufacturing of products with similar properties to those of the control cookies.

Coefficients of correlation of consumers rating with instrumental measurements of cookie structure (Table 4) were the highest (−0.95) between increasing hardness of cookies (due to rising proportions of potato pulp) and lowering scores of parameters like structure, cohesion, and taste and smell.

**Table 4:** Correlation coefficients between instrumental measurements of cookies and their consumer assessment

	Maximum force needed to cut a cookie (*F*_max_)	Energy needed to cut a cookie (*E*)
Cohesion	−0.96	−0.96
Structure	−0.95	−0.95
Taste and smell	−0.96	−0.96

Our findings are consistent with earlier studies. Baumgartner et al. [30] studied cookies with part of the flour replaced by oat bran. When 7–21% of flour was replaced by bran, no significant changes in cookie parameters were observed although their color was slightly darker. Curti et al. [31] investigated the influence of potato pulp on storage properties of bread and found an increase in product hardness during storage. Mudgil et al. [32] added guar gum to cookies as a fraction of soluble fiber. Those authors reported a slightly lower acceptability of the cookies with guar gum compared to that of control cookies (7.51 vs 8.00).

It can be concluded that the production of shortcrust pastry cookies with up to 40% of flour replaced by dried potato pulp can be recommended. Mechanical properties of such cookies and their acceptance by consumers are similar to those of control cookies. The potato pulp used in this study contains 52.7% total dietary fiber. It means that the use of the dried potato pulp can markedly increase the dietary fiber content of such products, from about 6% when 20% of flour replaced by dried potato pulp, up to 30% when wheat flour is completely replaced by the potato pulp. Menis-Henrique et al. and Schmid et al. stated that heat treatment does not significantly reduce the amount of fiber fraction [33,34], but several studies have revealed that the incorporation of high fiber components increased significantly the total dietary fiber in the product after exposure to high temperature [24,27,35]. Jeddou et al. reported that the incorporation of the potato peel powders in cakes at 10% lead to an increase in the content of both soluble and insoluble fiber [29]. In other studies, the increase in the dietary fiber after cooking [36,37], frying, or roasting of potato was observed [38]. The increase in the dietary fiber could generate the formation of resistant starch in products after thermal processes and the formation of complexes between polysaccharides and other compounds in the food (such as protein or lipid) [37,39].

## 4 Conclusions

The results of this study proved that potato pulp may be an excellent source of dietary fiber that can be used as a substitute of flour in cookies. Moreover, noteworthy is the fact that in this way, potato pulp is used as a source of fiber, and also this burdensome by-product of the starch industry can be utilized. The addition of potato pulp in an amount of up to 40% does not cause any remarkable change in their crispness and hardness, but shifts the color of the cookies from yellow towards green and causes a significant decline in their color saturation. It is a success that consumers rated the cookies with the addition of pulp in an amount up to 40% as acceptable, which means that a large amount of this waste can be utilized in the confectionery industry.
